# Novel species identification and deep functional annotation of electrogenic biofilms, selectively enriched in a microbial fuel cell array

**DOI:** 10.3389/fmicb.2022.951044

**Published:** 2022-09-14

**Authors:** Lukasz Szydlowski, Jiri Ehlich, Pawel Szczerbiak, Noriko Shibata, Igor Goryanin

**Affiliations:** ^1^Biological Systems Unit, Okinawa Institute of Science and Technology, Onna, Japan; ^2^Malopolska Centre of Biotechnology, Jagiellonian University, Krakow, Poland; ^3^Faculty of Chemistry, Brno University of Technology, Brno, Czechia; ^4^School of Informatics, University of Edinburgh, Edinburgh, United Kingdom; ^5^Tianjin Institute of Industrial Biotechnology, Tianjin, China

**Keywords:** microbial fuel cell, metagenome, function prediction, copper, printed circuit board (PCB), bioleaching

## Abstract

In this study, electrogenic microbial communities originating from a single source were multiplied using our custom-made, 96-well-plate-based microbial fuel cell (MFC) array. Developed communities operated under different pH conditions and produced currents up to 19.4 A/m3 (0.6 A/m2) within 2 days of inoculation. Microscopic observations [combined scanning electron microscopy (SEM) and energy dispersive spectroscopy (EDS)] revealed that some species present in the anodic biofilm adsorbed copper on their surface because of the bioleaching of the printed circuit board (PCB), yielding Cu2 + ions up to 600 mg/L. Beta- diversity indicates taxonomic divergence among all communities, but functional clustering is based on reactor pH. Annotated metagenomes showed the high presence of multicopper oxidases and Cu-resistance genes, as well as genes encoding aliphatic and aromatic hydrocarbon-degrading enzymes, corresponding to PCB bioleaching. Metagenome analysis revealed a high abundance of *Dietzia* spp., previously characterized in MFCs, which did not grow at pH 4. Binning metagenomes allowed us to identify novel species, one belonging to *Actinotalea*, not yet associated with electrogenicity and enriched only in the pH 7 anode. Furthermore, we identified 854 unique protein-coding genes in *Actinotalea* that lacked sequence homology with other metagenomes. The function of some genes was predicted with high accuracy through deep functional residue identification (DeepFRI), with several of these genes potentially related to electrogenic capacity. Our results demonstrate the feasibility of using MFC arrays for the enrichment of functional electrogenic microbial consortia and data mining for the comparative analysis of either consortia or their members.

## Introduction

Microbial fuel cells (MFCs) are a type of chemical fuel cell in which the anodic reaction is catalyzed by various microorganisms that oxidize organic matter. When coupled with the cathodic reduction of oxygen, this system yields energy in the form of electricity. Given the exponential growth of studies focused on MFCs and electrogenic bacteria in general (Santoro et al., 2017), numerous reactor designs have been developed. However, the singularities of these systems render the reproducibility of experiments extremely difficult. Prior focus on unifying reactor conditions to study extracellular electron transfer (EET) has been on manufacturing multiple stand-alone microbial reactors, such as those based on small glass vials (Call and Logan, 2011a,b). The reactor design, materials used, dimensions, and electrochemical properties (e.g., internal resistance) have differed between research groups. Hou et al. (2009, 2011, 2012) demonstrated the use of 24-well plate arrays comprising microfabricated gold electrodes with ferricyanide (Hou et al., 2009), air-cathodes (Hou et al., 2011), or microfluidic channels with continuous anolyte and catholyte replenishment (Hou et al., 2012), which increased the power output by a factor of three. These reactors were used to screen previously selected electrochemically active environmental isolates.

Another example of a well plate array implementation was demonstrated by Yuan et al. (2011), in which EET was coupled to the color change of the probe. Recently, Molderez et al. (2021) constructed a 128-channel potentiostat connected to a printed circuit board (PCB) microarray. The entire microarray was immersed in an anolyte solution and supplied with a reference electrode to perform a high-throughput investigation pertaining to the effect of the anodic potential on electroactive biofilm growth. Zhou et al. (2015) proposed a well-plate-based, high-throughput colorimetric assay for microbial electrochemical respiration to indicate EET. Alternatively, a 48-well plate with a hydrophobic wax layer separating electrodes was developed by Choi et al. (2015), followed by a single-sheet paper-based electrofluidic array (eight cells) developed by Gao et al. (2017). Tahernia et al. (2019) developed paper-based, disposable 64-well arrays yielding power densities of up to 23 μ*W/*cm^2^. The device has been successfully used to characterize the electrochemical properties of various *Shewanella oneidensis* and *Pseudomonas aeruginosa* strains. They later developed, a 96-well electrofluidic array using the same fabrication method (Tahernia et al., 2020). Recently, another 96-microwell device (four 24-well modules) was demonstrated, with three electrodes and gas outlets to maintain anaerobic environment (Kuchenbuch et al., 2022). These newly developed MFC platforms allow higher precision in comparative studies of electrogenic biofilms. Metagenomes derived from electrogenic communities encode many unique genes that help with EET. Moreover, metabolic pathways within electrogenic communities can specialize in the degradation of toxic compounds and precipitation of heavy metals, thereby offering energetically favorable alternatives to existing bioprocesses.

Metagenomic assembly and annotation have become increasingly informative with the constant growth of databases. For example, the number of reference human gut bacterial genomes increased from 194 in 2010 (Qin et al., 2010) to 204938 in 2021 (Almeida et al., 2021). Advances in sequencing technology now allow single genomes to be assembled directly from the metagenome, creating metagenome- assembled genomes (MAGs)(Tyson et al., 2004). Despite being more abundant than human microbiomes, environmental metagenomes are less resolved, with only 52,515 MAGs (Nayfach et al., 2021). MAGs have allowed for the discovery of novel metabolic pathways, such as commamox (Daims et al., 2015).

However, as the majority of MAGs are uncultured organisms, it is not surprising that a substantial proportion of genes lacks functional annotation. To make a comparative analyses of metagenomes easier, several tools for *de novo* annotation have been developed (Chen et al., 2020; Almeida et al., 2021; Beghini et al., 2021; Nayfach et al., 2021).

In this study, we present a 30-day enrichment of electrogenic consortia derived from a single inoculum [air-conditioning (AC) outflow] fed with an identical substrate. We applied different pH conditions to those consortia within identical reactors to compare their electrochemical performance in relation to changes in microbial community structure. We sampled anodic biofilms from all tested groups and visualized them under an electron microscope. Furthermore, we performed metagenomic sequencing and subsequent genome assembly and annotation from different compartments of the MFCs [anodes, cathodes, and open circuit potential (OCP) controls], which allowed for the identification of new species from the MAG isolates. Unannotated, unique genes present in novel electrogenic MAGs were further investigated using *in silico* analysis tools such as DeepFRI (Gligorijevic et al., 2021) and AlphaFold (Jumper et al., 2021).

## Materials and methods

### Microbial fuel cells construction and operation

**FIGURE 1:**
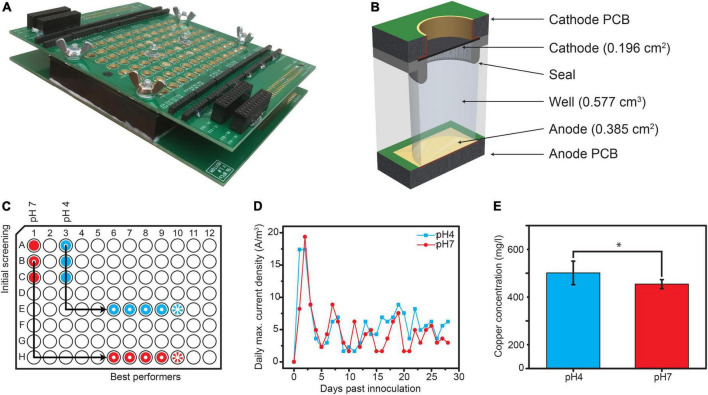
**(A)** Overview. **(B)** Cross-section of the used array. **(C)** Experimental setup, with the wells marked red being the pH 4 group, wells marked blue being the pH 7 group and wells with rays being the open circuit potential (OCP) controls. **(D)** Daily maximal current densities measured post inoculation. **(E)** Cu concentrations measured from the effluent samples of each well. Statistical significance was tested using ONE-way ANOVA (*p-value < 0.05).

Each well was built as an individual MFC with a membraneless design (Figure 1), as described previously (Szydlowski et al., 2022), with the following modifications: the base anode plate was built from a standard PCB comprising a thin copper sheet (17 μ*m*) with electroless nickel plating and covered with a thin layer of immersion gold (ENIG—RoHS by JLCPCB, China). The seal was manufactured from polydimethylsiloxane (Sylgard 184; Sigma Aldrich) using a custom-made mold. The volume of each well was 0.577 cm^3^ and the spacing between the electrodes was 1 cm. Samples from an AC unit outflow in Ishikawa, Japan (26.43°N, 127.84°E) were mixed (1:3) with basal medium containing 200 mg/L CaCl22H_2_O, 250 mg/L MgCl26H_2_O, 500 mg/L NH_4_Cl, trace elements, and vitamin solution (medium 141; DSMZ) containing 1 g/L acetate and 2 g/L lactate as the carbon source. The pH of the medium was adjusted to either 4 or 7 prior to sample addition and samples were incubated overnight at 25°C. To inoculate the 96-well plate, 15 carbon veil (7 g Elite Motoring; United States) disks (0.385 cm^2^ each) were immersed in each solution overnight and transferred to a 96-well plate (in stacks of five) as follows: disks incubated at pH7 were placed into wells A1-C1, and disks incubated at pH4 were placed into wells A4-C4. External resistors (R_*ext*_ = 330 Ω) were connected to the aforementioned wells, as determined by electrochemical impedance spectroscopy (EIS). The potential between the electrodes with attached external resistors was measured for 72 h, and the current was derived using Ohm’s law. The best-performing wells from each pH regime were then selected (Figure 1A) and multiplied as follows: disks from A4 were placed into wells E6-E10 (pH4), and disks from B1 were placed into wells H6-H10 (pH7). Wells E10 and H10 served as OCP controls for the pH4 and pH7 group, respectively.

External resistors of 330 Ω were attached to wells E6-E9 and H6-H9. An additional four disks were added to each well to allow for biofilm growth. The same medium was used throughout the experiment, and the 96-well plate was incubated on a bench at 25°C. The medium was replaced with the same volume of fresh medium (577 μl) daily by opening the top PCB. The samples were then subjected to volatile fatty acid (VFA) analysis using ion chromatography (IC). The top carbon sponges on the cathodes were washed daily with Milli-Q water. The potentials between electrodes with external resistors attached were measured for 4 weeks using PalmSens3, and the current was derived using Ohm’s law. The current density (*j*) was normalized to the volume of each well (0.577 cm^3^) or anode surface area (0.385 cm^2^).

### Electrochemical and chemical assays

Cyclic voltammetry (CV) and linear sweep voltammetry (LSV) measurements were performed using a PalmSens3. EIS was performed using a Gamry Interface 1,000 instrument. A two-electrode setup was used for measurements after 30 min left at the OCP, with anodes serving as the working electrodes and cathodes as the counter and reference electrodes. For CV measurements, the potential (E) range was from 0.4 V to –0.7 V and scan rate was 0.1 mV/s. For LSV measurements, the E range was from 0 mV vs. OCP to 0 mV vs. reference electrode, scan rate was 0.1 mV/s and ste was 0.2 mV. For EIS, the frequency range was between 1 and 50 MHz, with 7 mV steps. The copper concentration in the liquid samples was determined using inductively coupled plasma mass spectrometry (ICP-MS). Samples were diluted 100,000 × with Milli-Q water and treated overnight with 5% HNO3 to remove residual organic matter. Each sample was measured in triplicate, and analysis of variance (ANOVA) was performed for all samples.

### Scanning electron microscopy and energy dispersive spectroscopy analysis

Anodes were fixed by soaking in 2.5% glutaraldehyde for 12 h at 4°C. They were then washed three times with 0.1 M phosphate buffer of pH 7 at 4°C, dehydrated with a series of ethanol solutions (50, 70, 80, 90, 95, and three times in 100%). Next, the anodes were soaked in pure hexamethyldisilazane twice for 30 s, as previously described (Araujo et al., 2003). After drying for 10 min, the samples were sputter-coated with gold. The samples were observed by scanning electron microscopy (SEM) (JSM-7900F JEOL). Additionally, EDS scans were conducted to detect the presence of Cu in the biofilms.

### Metagenomic sequencing and analysis

DNA was extracted from the AC outflow, as well as from the carbon veil disks, from each well (E6-10, H6-10) using the Maxwell RSC kit and automated station and subjected to Illumina NovaSeq sequencing at the Okinawa Institute of Science and Technology (OIST) DNA sequencing facility. DNA samples derived from wells E6-9 and H6-9 were pooled prior to sequencing. The metagenomic sequences were processed using the KBase platform (Arkin et al., 2018). First, paired-end reads were subjected to quality control and filtering using FastQC v0.11.5. For phylogenetic analysis of the metagenomes, paired-end libraries were subjected to the Kaiju pipeline (Menzel and Krogh, 2016), and beta-diversity was computed using QIIME2 (Caporaso et al., 2010) and PALADIN (Westbrook et al., 2017) (with the Swiss-Prot reference database) using taxonomic and functional features, respectively and the Principal coordinate analysis (PCoA) was performed, using Bray-Curtis dissimilarity on a relative abundance matrix. Subsequently, metagenomic reads were assembled using metaSPAdes v3.13.0 (Nurk et al., 2017). For functional analysis, contigs were binned using MaxBin2 v2.2.4 (Wu et al., 2016), and the quality of the extracted genomes was assessed using CheckM v1.0.18 (Parks et al., 2015). Multiple assemblies were annotated using RASTtk (Brettin et al., 2015) and multiple domain annotation tools and subjected to comparative studies. Binned contigs were subjected to average nucleotide identity (ANI) analysis (Ciufo et al., 2018) and a phylogenetic tree was constructed using FastTree2 (Price et al., 2010). For all binned contigs from each metagenome set, sequence similarity analysis was performed using a search command with default parameters in the MMseqs2 package (Steinegger and Soding, 2017). Two compared sequences were annotated as being similar if they were simultaneously evaluated with an E-value ≤ 10^–5^ and a bit score ≥ 50. Sequence-based functional annotations were generated by DeepFRI using CPU models (Gligorijevic et al., 2021).

## Results

### Microbial fuel cells operation and electrochemical measurements

Our AC outflow consortium was inoculated into a matrix of wells, with pH and circuit being the two variables (Figure 1A). The initial screening revealed that two wells from each group maintained stable potentials, equivalent to current densities of 9 and 1.2 A/m^3^ in the pH 4 and pH 7 groups, respectively (Supplementary Figure 1A). Disks from these wells were used to inoculate new sets of wells. In the first 2 days post multiplication, current densities reached a maximum of 17.4 and 19.4 A/m^3^ in the pH 4 (well E6) and pH 7 (well H8) samples, respectively. Then, the currents decreased and kept oscillating between 1.6 and 8.8 A/m^3^ in the following days, with the pH4 wells showing slightly higher values than pH 7 (Figure 1B). In terms of power, our MFCs produced up to 30.1 and 80.2 nW (which is equivalent to 0.8 and 2.1 μW/m^2^ or 52.2 and 139 mW/m^3^) in wells E6 (pH 4) and H8 (pH 7), respectively (Supplementary Figure 2). Based on the IC analysis, the total chemical oxygen demand (COD) removal was 1996 ± 127 mg COD/L/d and 1806 ± 137 mg COD/L/d for the pH 4 and pH 7 samples, respectively (Supplementary Figure 1B). The pH increased from 4 to 7 in wells E6-E9 and from 7 to 10 in wells H6-H9 after 24 h.

CV scans in the pH 4 blank reveals a smooth curve non-Faradaic current of 30 μ*A* and a small reduction peak at 0.55 V. Two weeks post inoculation, an oxidative peak was seen at 0.13 V, with an oxidative wave at 180 μ*A* and a reductive peak at –0.44 V with a reductive wave below –200 μ*A* (Supplementary Figure 2A). In the pH 7 samples, the non-Faradaic currents were below 30 μ*A*, with no redox peaks. Two weeks after inoculation, the oxidative peak was approximately 0 V, with an oxidative wave at 80 μ*A* and a reductive peak was –0.25 V with a reductive wave at –170 μ*A* observed (Supplementary Figure 2B). Thus, the capacitance increase was over 12-fold and eightfold in the pH 4 and pH 7 MFCs, respectively, indicating the presence of a biofilm on our electrodes.

#### Copper presence and inductively coupled plasma mass spectrometry measurements

Two days after inoculation, we identified copper ions in the anolyte outflow of the pH 4 samples, potentially derived from PCB. We subjected outflow samples to ICP-MS and determined the average concentration of copper to be 510 ± 63 and 450 ± 24 mg/L for the pH 4 and pH 7 samples, respectively (Figure 1E), whereas the Cu^2+^ concentration in the prepared media was below the quantification limit (3 μg/L). ANOVA revealed a statistical difference in the Cu^2+^ concentration between the different pH groups.

#### Scanning electron microscopy and energy dispersive spectroscopy analyses

**FIGURE 2:**
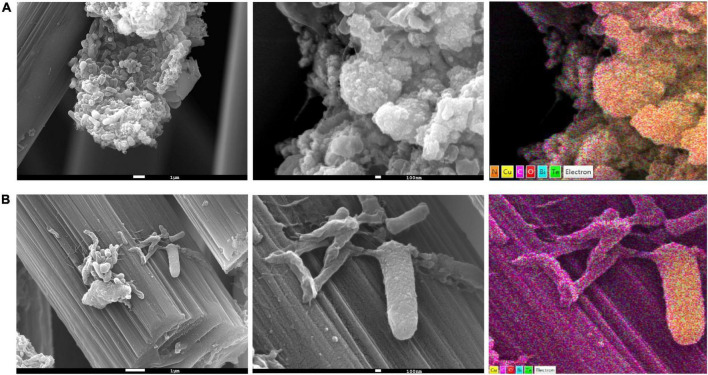
Scanning electron microscopy (SEM) and energy-dispersive spectroscopy (EDS) elemental mapping of anodic biofilms from the **(A)** pH 4 **(B)** and pH 7 electrodes.

SEM images of the anodic biofilms clearly indicated that the particles were adsorbed on the cell surfaces. When further analysis was performed using an EDS detector, a strong signal was obtained from copper (Figure 2, right panel). Moreover, in some parts of the biofilm, Cu^2+^ was present only on some specimens, as the Cu signals from the arbon fibers and other parts of biofilm were much weaker (Supplementary Figure 3), suggesting that the community comprised members that actively sequestered Cu ions from the solution.

### Metagenomic analysis

#### Taxonomy analysis and beta-diversity

**FIGURE 3:**
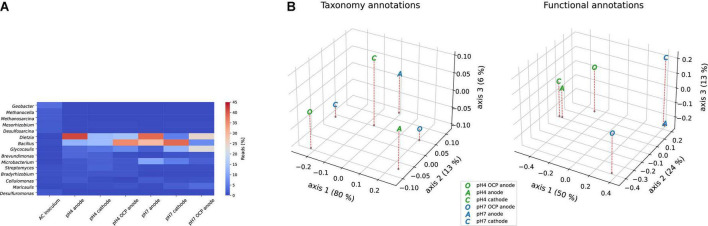
**(A)** Taxonomy analysis of the top 15 genera across all metagenomes. **(B)** Principal coordinate analysis (PCoA) based on taxonomy (genus level) and functional features of the metagenomes using Bray-Curtis dissimilarity on a relative abundance matrix.

Taxonomy profiles of our metagenomes revealed that *Dietzia* spp. was the most dominant anodic genus in both the pH 4 and 7 reactors (Figure 3A). Other abundant genera detected in the samples were *Bacillus*, *Glycocaulis*, and *Microbacterium* spp., which were also abundant in the OCP controls. In the pH 4 and pH 7 OCP controls, the most abundant genera were *Bacillus* and *Dietzia/Glycocaulis*, respectively, whereas the cathodes comprised mostly of *Bacillus* in both pH groups. In the AC outflow, *Geobacter* spp. was the most abundant genus, with 4% abundance. *Dietzia* comprised only 0.04% of the inoculum. PCoA was performed using the taxonomy and functional features (Figure 3B) of enriched metagenomes. In terms of taxonomy, divergence was observed across all samples. However, when functional annotations were compared, clusters based on pH were observed, with the pH 4 anode and cathode being closer than their corresponding pH 7 metagenomes. The OCP controls from each of the groups diverged from the other samples, indicating that the electric circuits influenced biofilm metabolism.

#### Metagenome assembly and annotation

All metagenomes were then assembled using MetaSPAdes (Nurk et al., 2017), and the resulting contigs were annotated using Prokka (Seemann, 2014) and RAST (Brettin et al., 2015). A comparative analysis of the annotated contigs was performed, and the set intersections were aggregated, as shown in Supplementary Figure 4. The highest number of common annotations was found in all metagenomes except the pH 4 cathode (2419), whereas all metagenomes shared 709 common annotations. The entire pH 4 group (anode, cathode and OCP) had 959 common annotations, whereas the entire pH 7 group had 436 annotations. For the pH 4 and pH 7 anodes and cathodes, the number of common annotations was below 10, and control anodes (O) had only 10 common annotations. The highest number of unique annotations present in a single metagenome was in the pH 7 OCP sample. In contrast, the pH 4 OCP had the lowest number of unique annotations (35). The anodes showed 72 and 69 unique annotations in the pH 4 and pH 7 groups, respectively, whereas the cathodes showed 43 and 657 unique annotations in these groups. All annotation data are presented in Supplementary Table 1. As expected, all metagenomes also contained various Cu-related genes (Table 1), such as multicopper- and heme-copper oxidoreductases, metallochelatins, ATP-dependent heavy metal translocases, genes indicating Cu resistance (e.g., cupredoxins, *cop*- and *cut*-family resistance genes), copper-sensing two-component system response regulator *cpx*R, and the *cso*R repressor of the *cop*ZA operon. All metagenomes shared similar numbers of these genes (52–74), except for the pH 4 cathode, where only seven Cu-related annotations were found. Moreover, all contigs contained genes involved in electron transfer pathways, with two to three times more genes in the pH 7 anode community than the pH 4 counterparts. Notably, genes encoding the nitrogenase FixABCX protein complex were found in all metagenomes, but six *rnf* genes were found exclusively in the pH 7 OCP metagenome. In agreement with PCB bioleaching, as indicated by the presence of dissolved Cu, genes involved in the degradation of various hydrocarbons (both aromatic and aliphatic) were also found, with higher gene counts observed in the pH 7 group.

**TABLE 1:** Gene count and abundance for the three pathways in all metagenomes.

Genes	pH4 A	pH4 OCP	pH4 C	pH7 A	pH7 OCP	pH7 C
Cu-related	65	68	7	74	74	52
*Cu-related abundance*	1.35%	1.22%	0.36%	1.33%	1.14%	0.99%
Electron transfer	10	11	4	21	34	11
*Electron transfer abundance*	0.21%	0.20%	0.21%	0.38%	0.52%	0.21%
Hydrocarbon degradation	2	2	1	4	8	4
*Hydrocarbon degradation abundance*	0.04%	0.04%	0.05%	0.07%	0.12%	0.08%

**FIGURE 4:**
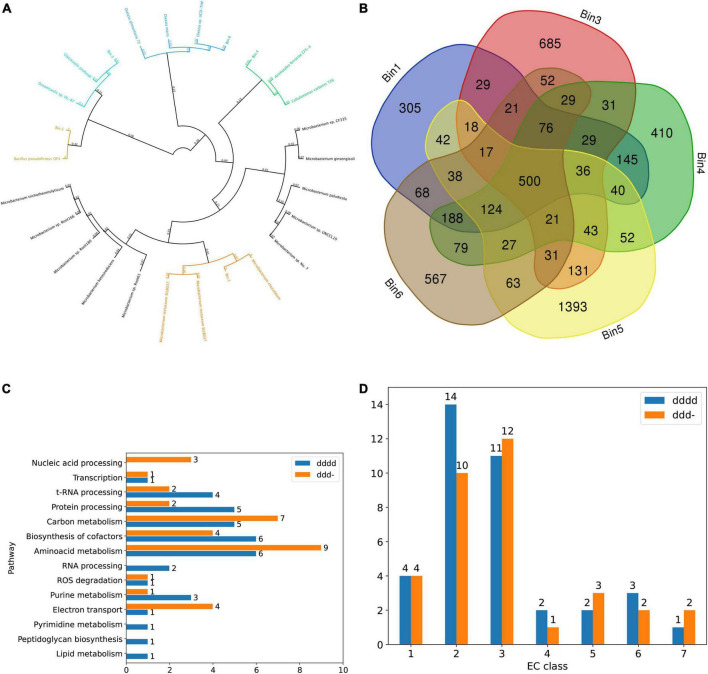
**(A)** Phylogenetic tree of bins from the pH7 anodes (numbers indicate substitutions per site). **(B)** Venn diagram showing common annotations. **(C)** The number of Bin.4-unique genes in different metabolic pathways. **(D)** The number of Bin.4-unique genes across EC classes for two deepest levels of EC classifications (dddd and ddd- respectively).

Binned contigs from pH 7 anode that revealed high quality (> 95% completeness and < 2% contamination) MAGs were annotated as *Microbacterium* (Bin.1), *Maricaulaceae* (Bin.3), *Actinotalea* (Bin.4), *Bacillus* (Bin.5) and *Dietzia* spp. (Bin.6; Figure 4A). Among these MAGs, all bins except Bin.5 were identified as novel species, according to the phylogenetic analysis and ANI check (Supplementary Table 2). However, the aforementioned MAGs were found in both pH4 and pH7 anodes and OCP controls, except for Bin.4, which was found exclusively in the pH 7 anode, and its abundance in the initial inoculum was less than 0.002%. All binned MAGs were subjected to domain annotation using Prokka and RAST (Supplementary Table 3). All pH7 bins had 500 common annotations, whereas MAG-unique annotations varied from 305 to 1,393 in Bin.1 and Bin.5, respectively (Figure 4B), which corresponds to their genome sizes (3,137 and 4,744 genes in Bins 1 and 5, respectively). Within the shared group, several TRX-like ferredoxin, Ni-Fe and Fe-only hydrogenases, and Fe ion transporters were identified. All binned contigs shared genes encoding enzymes for degradation of various hydrocarbons (both aromatic and aliphatic) and steroids. All contigs also contained various Cu-related genes, such as multi- and heme-copper oxidoreductases and ATP-dependent heavy metal translocases, whereas genes indicating Cu resistance (e.g., cupredoxins, copper-resistance genes *copA/C/Z* or metallochelatins were not found in all MAGs, indicating community interactions and complementarity. Apart from the genes with known annotations, our focus was also on the unknown genes that contributed to a large portion of all MAGs and metagenomes. Given that the *Actinotalea* MAG was found exclusively in the pH 7 anode community, we performed a comparative analysis of MAGs within the pH 7 anode and identified 854 unique (showing e-value > 10^–5^ and bitscore < 50) open reading frames (ORFs) when aligned against other metagenomic sequences, which was the highest value for either annotated or unannotated ORFs. Next, we analyzed the potential functions of these unique genes using DeepFRI. Using the enzyme commission (EC) classification, we identified genes encoding enzymes present in each EC class with the highest DeepFRI score assigned (Figure 4D) and divided them into metabolic pathways (Figure 4C). The number of unique genes reflects the redundancy of a specific EC class, with an EC2 (transferases), and EC3 (hydrolases) being the most numerous in Bin.4. These EC classes are generally the most abundant in all living organisms. In the case of transferases, we identified genes encoding virus-specific RNA-directed RNA polymerase (EC 2.7.7.48), indicating the presence of prophage elements.

**FIGURE 5:**
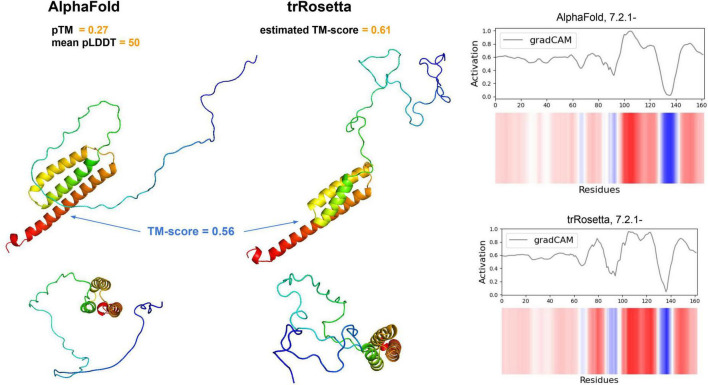
Alphafold (left) and trRosetta models (middle) of our putative reductase gene (EC 7.2.1.-). Each structure is shown from two different perspectives (lower and upper panels). The TM-score between both structures (excluding disordered tails) is depicted in blue. For both the AlphaFold and trRosetta predictions, the model quality measures are shown (see Yang et al., 2020; Jumper et al., 2021 for details). On the right panel, the DeepFRI activation map across residues is shown.

We especially focused on pathways that may play a key roles in the MFC environment: electron transfer (reductase, EC 7.2.1.-), reactive oxygen species (ROS) degradation (superoxide dismutase, EC 1.15.1.1), and cofactor biosynthesis (EC 2.5.1.78 involved in the flavin pathway and EC 4.2.1.96 involved in the quinone pathway). We processed ale these sequences using the trRosetta and AlphaFold algorithms to predict their structure, however, only one EC 7.2.1.- modeled with sufficient confidence (pTM score; Figure 5). In total, four Bin.4-unique genes encoding reductases (EC 7.2.1.-) were identified within the electron transfer pathway. This class contains three groups of enzymes: NAD + :ubiquinone (7.2.1.1), ferredoxin-NAD + (7.2.1.2) and ascorbate ferrireductase (7.2.1.3), which are all involved in the electron transfer pathway.

## Discussion

Our analysis identified the novel electrogenic bacterium *Actinotalea* sp. nov. has been identified in the anodic community of our MFC. *Actinotalea* is a high GC, gram-positive, facultative anaerobic genus derived from *Cellulomonas*; the main taxonomic difference is the major respiratory quinone menaquinone, with MK-10(H4) in the former and MK-9(H3) in the latter (Yi et al., 2007; Jin et al., 2017). *Actinotalea* can survive across a wide pH spectrum (4–11), although the most optimal conditions for its growth was pH 7 in our test. Other species from this genus have been found in soil (Suman et al., 2014; Yan et al., 2018), iron mine (Li et al., 2013), and biofilm reactors (Jin et al., 2017). The presence of numerous sequences related to electron transfer in its genome, e.g., the type IV pilus biosynthesis gene *pilB*, confirmed that this organism can respire through anodes, just like the well-studied electrogen *Geobacter* (McCallum et al., 2017). It would be interesting to compare the expression levels of the *Actinotalea*’s unique genes while grown in the MFC wrt OCP. Moreover, the presence of novel and unique enzymes, such as NADH translocases, could provide resilience to high Cu content. Many of the unannotated sequences obtained from the *Actinotalea* MAG received medium DeepFRI prediction scores, which may indicate the presence of a novel homologue; however, this must be experimentally validated.

Overall, the metagenomes analyzed in this study contained many genes encoding pathways related to anaerobic respiration. The inoculum derived from the AC outflow also consisted of marine bacterial species originating from the deep sea. Abrevaya et al. (2015) previously found *Dietzia* to be enriched in MFCs, although it is mostly found in oil-contaminated marine environments (Yumoto et al., 2002). Interestingly, despite its alleged alkalinity, we found the abundance of *Dietzia* to be similar in both pH 4 and pH 7 anodic samples (Figure 3), and binned contigs from both samples revealed 99.9% sequence similarity between the MAGs. This suggests that *Dietzia* can withstand periods of lower pH, since the pH increased from 4 to 7 in our pH 4 group. Moreover, its abundance in anodic biofilms may be explained by its ability to degrade numerous hydrocarbons (Yumoto et al., 2002). Owing to bioleaching of the PCB, Cu together with the organic epoxy coating become oxidized, thus creating compounds toxic to many bacteria. Since *Dietzia* can degrade these compounds, its abundance in anodic consortia is not surprising. The high abundance of EET-related and hydrocarbon degrading genes in pH 7 OCP, as seen in Table 1, as well as the total number of unique annotations may reflect higher metabolic diversity of this community due to the lack of external electron circuit. Indeed previous studies comparing MFC communities (Ishii et al., 2018; Kouzuma et al., 2018; Szydlowski et al., 2020) reveal higher functional diversity in communities grown at OCP, whereas the presence of external circuit applies selective pressure on the microbial metabolism.

Despite our efforts to separate the PCB from electrogenic bacteria, our results revealed that chemically deposited Au on the electrodes did not provide a sufficient barrier for the PCB and ultimately led to its corrosion. Such problems could be avoided by using PCBs with the so-called hard gold finish (electrochemically plated gold) or by covering the PCB anode with other conductive materials, such as stainless steel sheets, thus protecting the underlying Cu from being oxidized. Indeed, the electrochemical plating of the PCB successfully prevented Cu leakage in our modified well plate (Szydlowski et al., 2022). Owing to this unexpected leakage of Cu ions, we successfully enriched the Cu-resistant communities using our 96-well platform. Through SEM analysis, we observed that the anodic biofilm comprised species that concentrated Cu on their surfaces, leaving other parts of the biofilm relatively Cu-free. Therefore, it is tempting to suggest that this mechanism can be mediated by direct interspecies EET or electron bifurcation using Cu ions, as in the FixABCX (Ledbetter et al., 2017) or Rnf (Kuhns et al., 2020) complexes. Especially Rnf-encoding genes may be particularly interesting since unique genes were identified in the *Actinotalea* MAG that are putative members of the *rnf* family (Figure 5). Rnf enable difficult reductions (reviewed in Buckel and Thauer, 2018), which may indicate the ongoing degradation of the PCBs’ organic compounds, such as epoxy resins. Based on the studies describing Cu-bioleaching from PCB waste (Yang et al., 2009; Pant et al., 2012; Wu Y. et al., 2018), it can be concluded that Cu mobilization from its zero-valent state may be due to the bioelectrochemical cycle, which is also depicted in the model proposed by Becci et al. (2021). Copper can be usually oxidized by the simultaneous reduction of Fe^3+^ to Fe^2+^ and its efficiency depends on the reoxidation of iron back to Fe^3+^. Iron oxidation may be catalyzed by bacteria in the presence of oxygen, as well as alternative electron acceptors, such as electrodes, which in turn may explain the high current densities observed in our reactors. Moreover, simultaneous proton consumption that accompanies Fe reoxidation (Becci et al., 2021), can explain the pH shift observed in our study, as well as the differences in Cu content (Figure 1E). Wu W. et al. (2018) demonstrated a bacterial-free supernatant derived from Fe/S-oxidizing bacteria, also known for their electrogenic activity, resulting in complete Cu recovery from wasted PCBs. Other studies on the metallurgic, Cu-removing MFCs demonstrated that electrogenic consortia can reduce Cu from aqueous solutions and precipitate it on electrodes. The efficiency of this process depends on various factors, such as the use of pH separators (membranes), presence/absence of oxygen, and initial copper concentration (Heijne et al., 2010; Tao et al., 2011a,b; Motos et al., 2015; Miran et al., 2017). Given that Cu was cycling from solid state (PCB) to the solution and precipitated back to the anodic biofilm, the microbial communities enriched in our well plate exhibit a combination of oxidative and reductive bioleaching (Brar et al., 2021), which may also indicate the fluctuations in the current densities (Figure 1D).

Our results illustrate the extent of selective enrichment of our MFC array, which yielded electrogenic microbial communities capable of adapting to different physical conditions, such as pH and electric circuits, and resulted in bioleaching of the PCB. Through a combination of phylogenetic analysis and metagenome binning with *in silico* functional assays, we were able to identify, characterize and compare the microbial communities and link their features to the electrochemical performance that was measured in unified array conditions. This platform is a rapid, high-throughput system that offers parallelization for the screening of electrochemical microorganisms, as well as comparative analysis of functional metagenomes.

## Data availability statement

The datasets presented in this study can be found in online repositories. The names of the repository/repositories and accession number(s) can be found below: https://narrative.kbase.us/narrative/62324, 62324/1/362/.

## Author contributions

LS conceived the experiment(s). LS, JE, and NS conducted the experiment(s). LS and PS analyzed the results. All authors reviewed the manuscript and contributed to the article and approved the submitted version.
