# Supplementary material for: Novel species identification and deep functional annotation of electrogenic biofilms, selectively enriched in a microbial fuel cell array
Source: Front Microbiol. 2022 Sep 14;13:951044. doi: 10.3389/fmicb.2022.951044 (PMC9517587; doi:10.3389/fmicb.2022.951044)
Supplement: Supplementary file 1 [file Data_Sheet_1.zip › Data Sheet 1/Supplemetary file overview.docx]

**Novel species identification and deep functional annotation of electrogenic biofilms, selectively enriched in microbial fuel cell (MFC) array**

Supplementary figures:

**Supplementary Figure 1**. a) Daily current densities in the first 2 days past inoculation in pH 4 and pH 7 groups; b) Average COD removal (mg/L/d).

**Supplementary Figure 2**. Linear Sweep Voltammetry (LSV) -derived power curves (top) and Cyclic Voltammetry (bottom) scans in a) pH 4 and b) pH 7 groups.

**Supplementary Figure 3**. EDS atomic content analysis of electrode biofilm (anode pH 7).

**Supplementary Figure 4**. Upset plot indicating common annotations between all annotated metagenomes.

Supplementary Tables:

**Supplementary Table 1**. Annotations of all metagenomic contigs.

**Supplementary Table 2**. Average nucleotide identity of pH 7 anode bins.

**Supplementary Table 3**. Annotations of pH7 anode binned MAGs.
