# Supplementary figures and images for: Novel species identification and deep functional annotation of electrogenic biofilms, selectively enriched in a microbial fuel cell array
Source: Front Microbiol. 2022 Sep 14;13:951044. doi: 10.3389/fmicb.2022.951044 (PMC9517587; doi:10.3389/fmicb.2022.951044)

a)

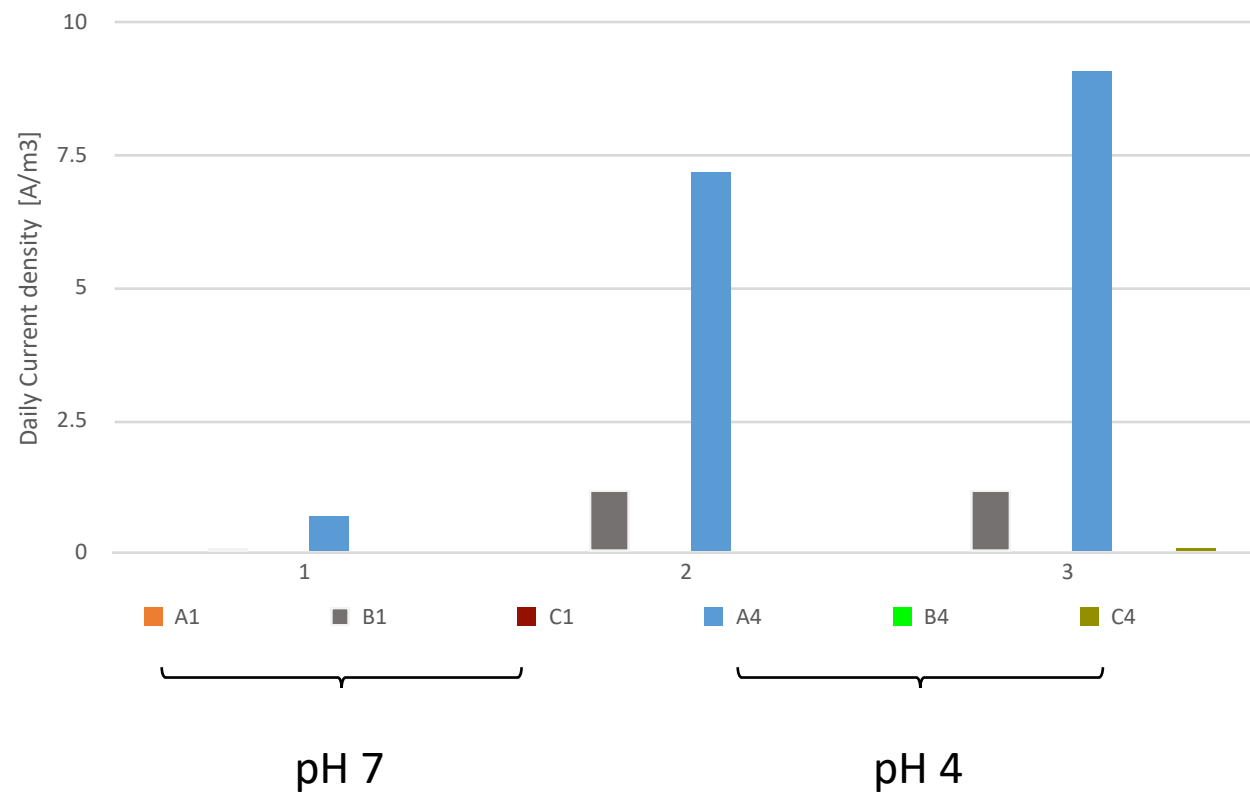

b)

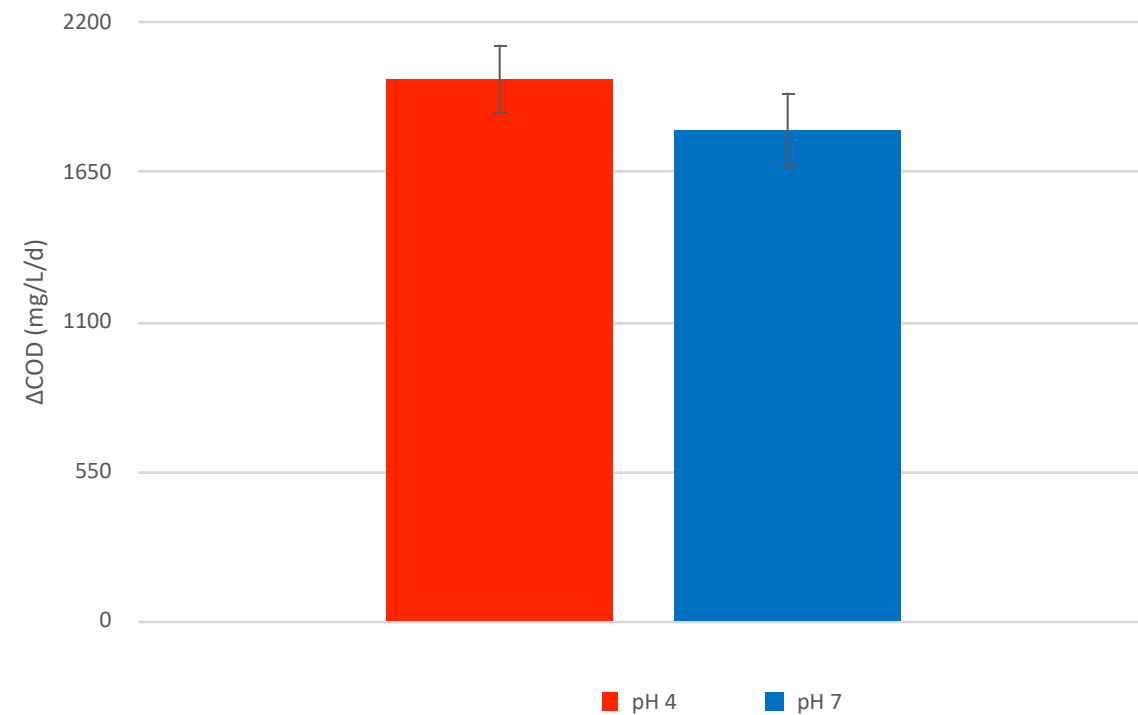

Supplement: Supplementary file 1 [file Data_Sheet_1.zip › Data Sheet 1/Figure S1.pdf]

a)

E6 (pH 4)

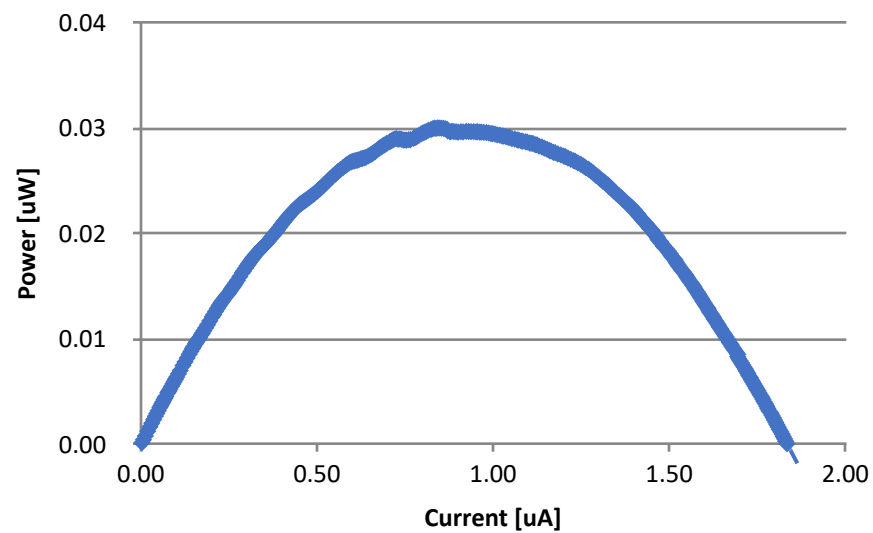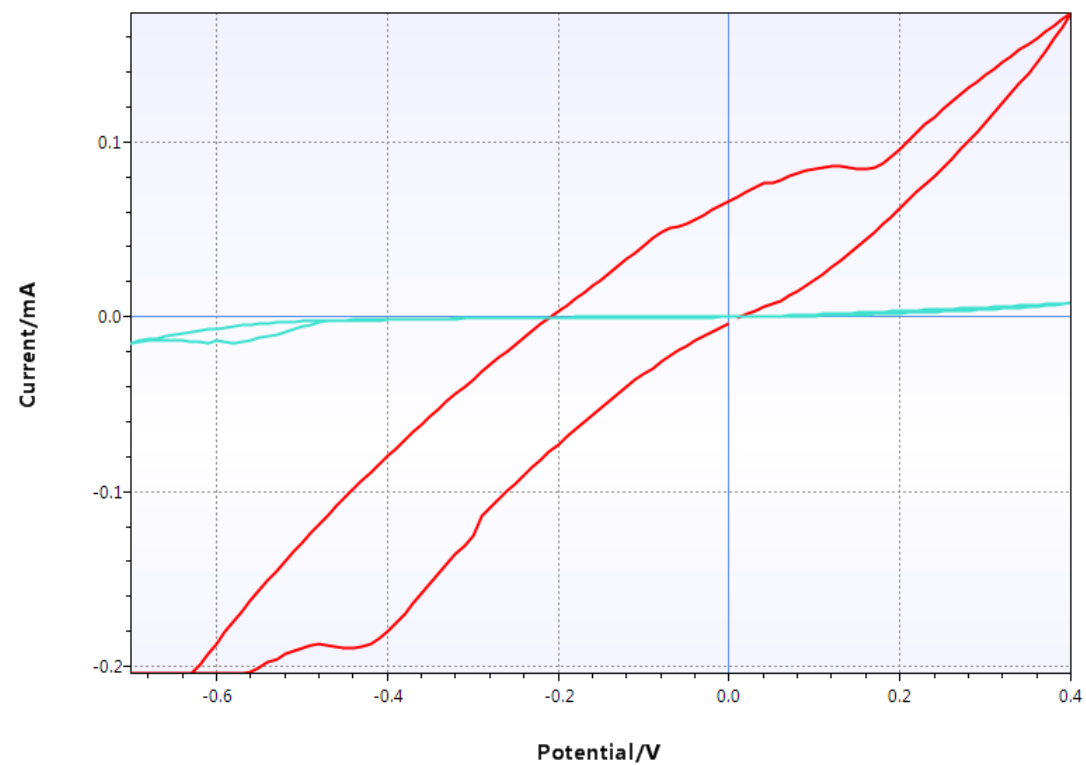

b)

H8 (pH7)

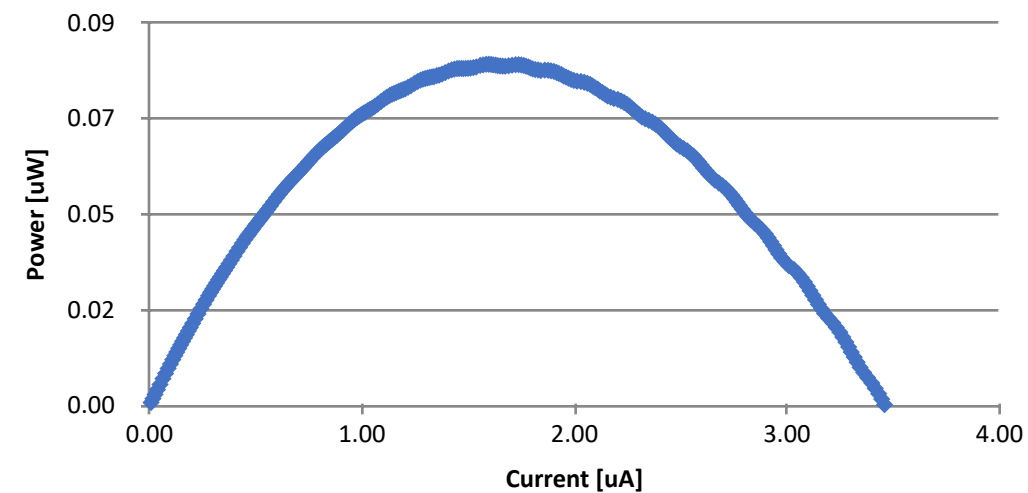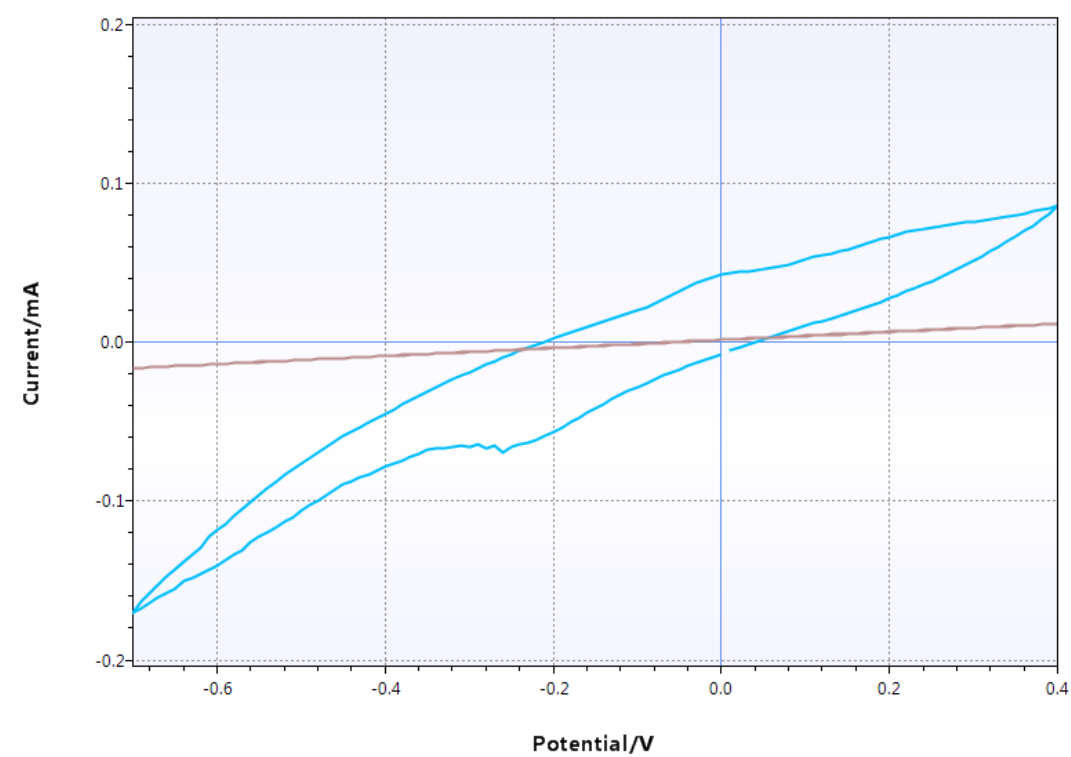

Supplement: Supplementary file 1 [file Data_Sheet_1.zip › Data Sheet 1/Figure S2.pdf]

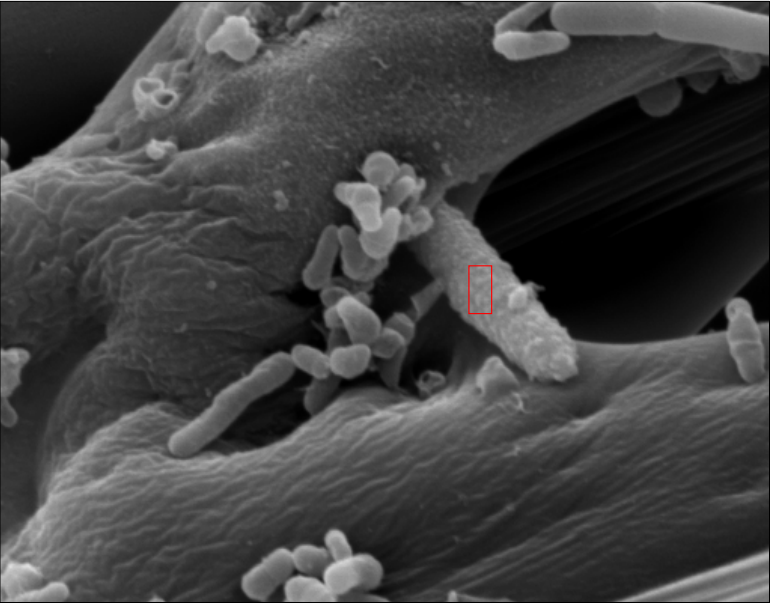

SE1 1μm

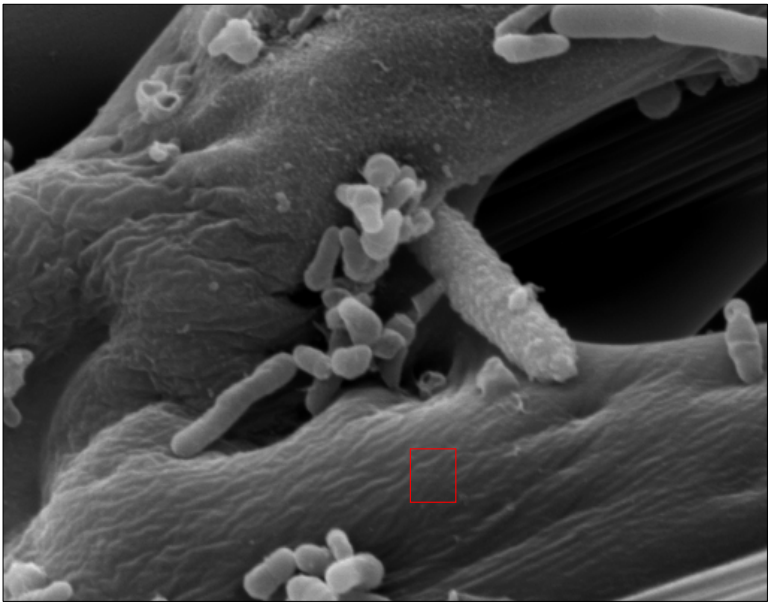

SE1 1μm

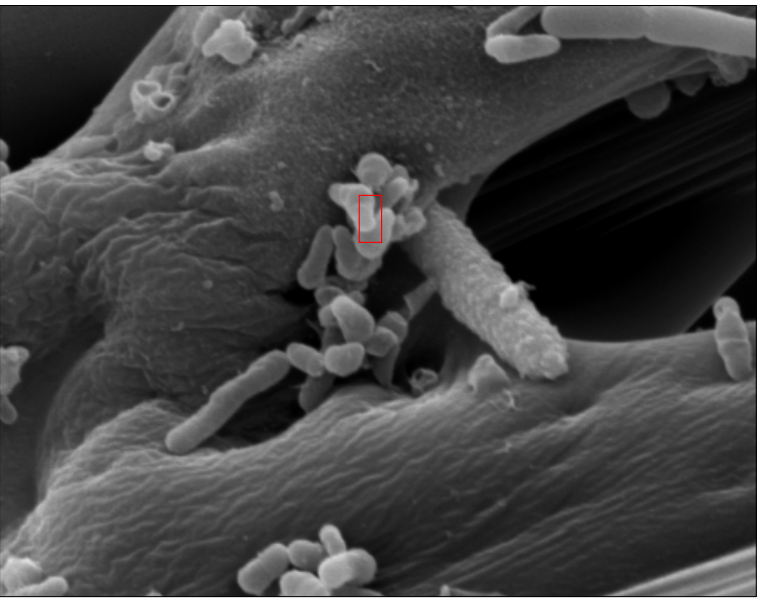

SE1 1μm

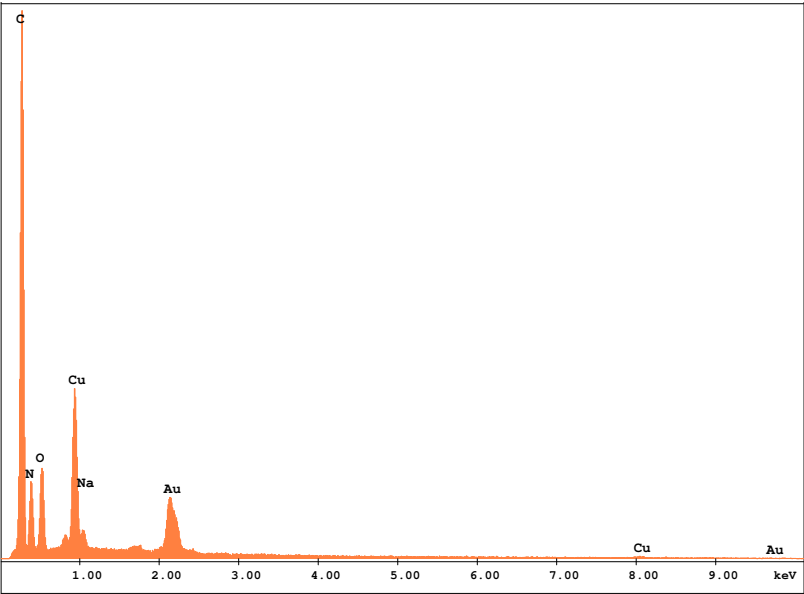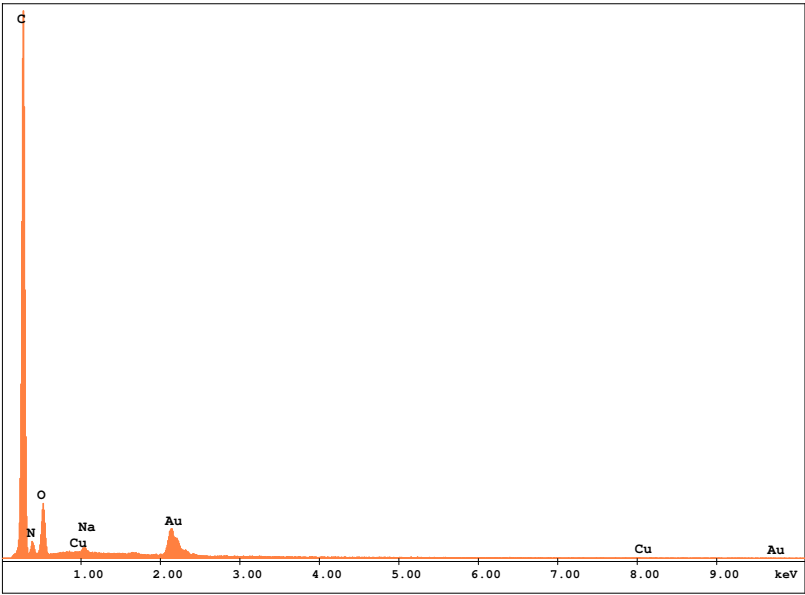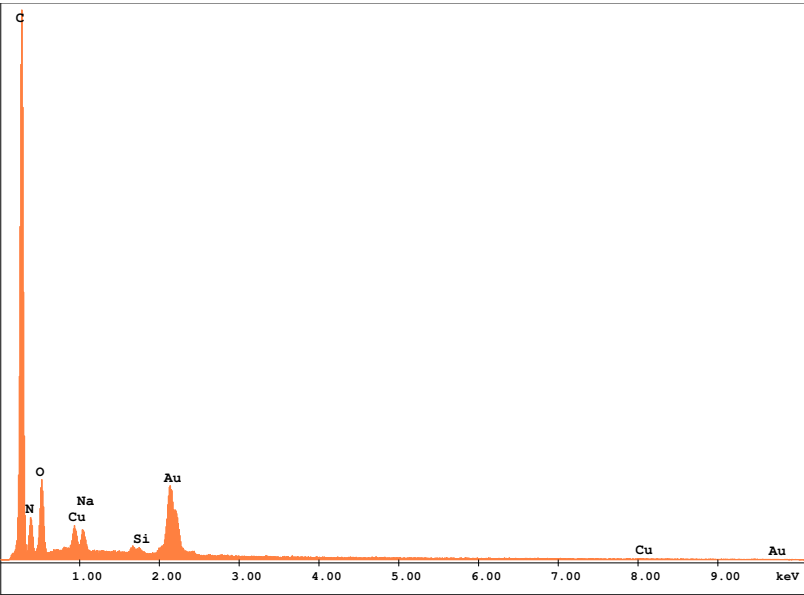

Supplement: Supplementary file 1 [file Data_Sheet_1.zip › Data Sheet 1/Figure S3.pdf]
