# Supplementary material for: Novel species identification and deep functional annotation of electrogenic biofilms, selectively enriched in a microbial fuel cell array
Source: Front Microbiol. 2022 Sep 14;13:951044. doi: 10.3389/fmicb.2022.951044 (PMC9517587; doi:10.3389/fmicb.2022.951044)
Supplement: Supplementary file 1 [file Figure S4.pdf › Data Sheet 1/Figure S4.pdf]

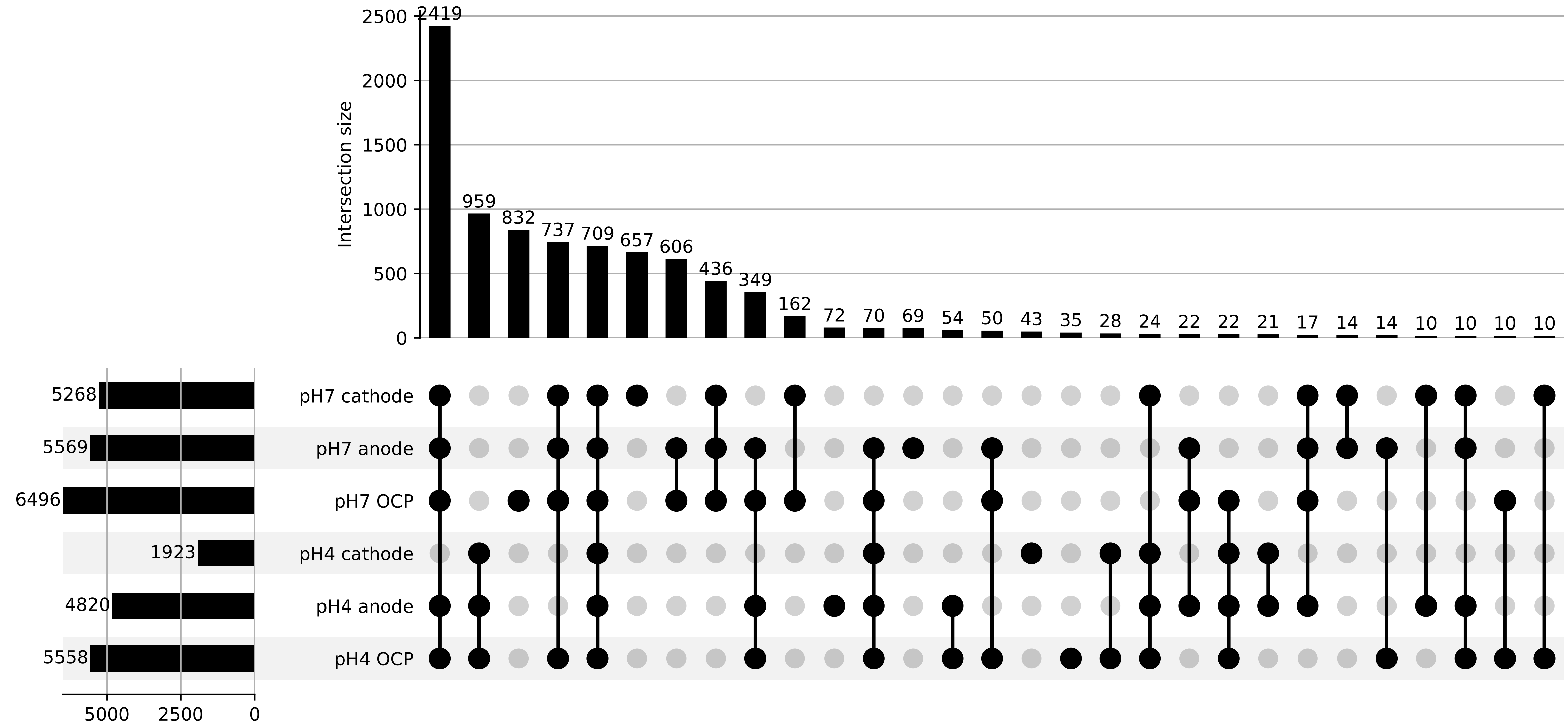

Intersection size
2500
2000
1500
1000
500
0
2419
959
832
737
709
657
606
436
349
162
72
70
69
54
50
43
35
28
24
22
22
21
17
14
14
10
10
10
10
pH7 cathode
pH7 anode
pH7 OCP
pH4 cathode
pH4 anode
pH4 OCP
5268
5569
6496
1923
4820
5558
5000
2500
0
